# Brodifacoum contamination of synthetic cannabinoid causing unexplained coagulopathy in multiple trauma: A case report

**DOI:** 10.1016/j.tcr.2024.101007

**Published:** 2024-04-01

**Authors:** Anthony V. Thomas, Mackenzie L. Johnson, Anna M. Tincher, Saniya Zackariya, Hassaan Khan, Uzma Rizvi, Scott G. Thomas, Timothy W. Noveroske, Daniel H. Fulkerson, Ernest E. Moore, Mark M. Walsh

**Affiliations:** a Indiana University School of Medicine, Indianapolis, IN, USA; b Departments of Emergency and Internal Medicine, Saint Joseph Regional Medical Center, Mishawaka, IN, USA; c Department of Mortality/Quality & Safety Data Review, Beacon Health System, South Bend, IN, USA; d Beacon Medical Group Trauma & Surgical Research Services, South Bend, IN, USA; e Department of Surgery, Saint Joseph Regional Medical Center, Mishawaka, IN, USA; f Department of Neurosurgery, Beacon Medial Group, South Bend, IN, USA; g Department of Surgery, Ernest E. Moore Shock Trauma Center at Denver Health, Denver, CO, USA

**Keywords:** Synthetic cannabinoids, Brodifacoum, Multiple trauma, Traffic accidents, Coagulopathy, Thromboelastography

## Abstract

An 18-year-old female presented to the emergency department after a motor vehicle collision. Initial imaging revealed a liver laceration. Subsequent labs showed significantly elevated prothrombin time, international normalized ratio, and activated partial thromboplastin time. Thromboelastography demonstrated a flatline tracing. The patient denied use of anticoagulation but admitted to synthetic cannabinoid use. It was believed the patient had taken synthetic cannabinoid contaminated by brodifacoum. She was therefore given prothrombin complex concentrate and vitamin K with blood products. The patient underwent sequential embolization, laparotomy, thoracotomy, and repair of the vena cava with a shunt. Thirty minutes postoperatively, her coagulation tests and thromboelastography were much improved. Two and a half hours postoperatively, it was determined she had sustained non-survivable injuries. The patient experienced brain death due to prolonged hypotension as a result of hemorrhagic shock with bleeding exacerbated by brodifacoum.

To our knowledge, this is the first case reported of a trauma-induced coagulopathy exacerbated by brodifacoum-contaminated synthetic cannabinoid. Her coagulopathy was clearly not due to trauma alone and contributed greatly to the difficulty in controlling hemorrhage. The synthetic cannabinoid-associated coagulopathy rendered her otherwise potentially survivable injuries fatal. Given the frequency of multiple trauma and the recent increase in the prevalence of synthetic cannabinoid, it can be expected that the incidence of trauma complicated by synthetic cannabinoid-associated coagulopathy will increase in the near future. For patients that present with prolonged prothrombin time and/or activated partial thromboplastin time, it is important to inquire about recent synthetic cannabinoid use.

## Highlights

• Brodifacoum is a vitamin K antagonist and an adulterant of synthetic cannabinoid.
• Synthetic cannabinoid contaminated with brodifacoum can contribute to coagulopathy.
• First case report of synthetic cannabinoid-associated coagulopathy in trauma.
• It is important to ask about synthetic cannabinoid use for prolonged PT/aPTT.
• Classical 1:1 mixing studies will show correction of PT/aPTT in these patients.

## Introduction

Brodifacoum is a powerful vitamin K epoxide reductase antagonist that is 100 times more potent than warfarin. The vitamin K antagonism can persist for an extended time with Brodifacoum measurable in the plasma for weeks and in the liver for months. It has been used as an adulterant in the preparation of synthetic cannabinoid (SC) [[1], [2], [3], [4]]. A recent review of 134 cases of SC intoxication not associated with trauma revealed coagulopathy in 34 patients which was rapidly corrected with prothrombin complex concentrate (PCC) and vitamin K [1]. Although the descriptions of synthetic cannabinoid-associated coagulopathy (SCAC) have increased recently, the syndrome of “superwarfarin”-induced coagulopathy is not new [2,3]. To our knowledge, this is the first case reported of a trauma-induced coagulopathy exacerbated by contamination of SC by brodifacoum.

## Case presentation

**Fig. 1:**
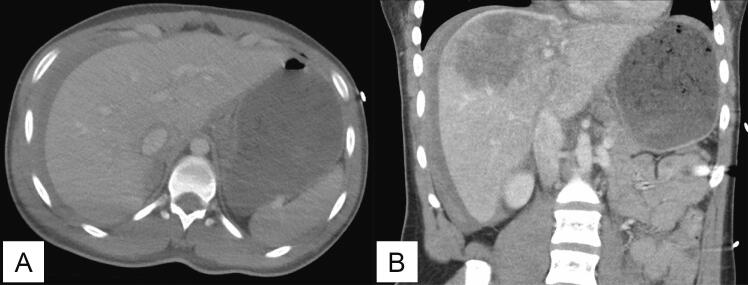
Transverse (A) and coronal (B) views of the patient's liver laceration pre-embolization demonstrating hemoperitoneum.

An 18-year-old female was involved in a head-on motor vehicle collision with an extrication time of 35 min. The patient was transported to a local hospital where she was stabilized. Computed tomography scans revealed a complex grade V liver laceration with hemoperitoneum and posterior dislocation of the right hip (Fig. 1). She was transferred to a level II trauma center by helicopter. She had received four units of whole blood and one liter of crystalloid by the time she arrived.

Upon arrival, she was awake with a Glasgow Coma Scale of 13. Her vital signs were heart rate 103 beats/min, respiratory rate 37 breaths/min, blood pressure 91/53 mmHg, and O_2_ saturation 99% on 100% oxygen. Her abdomen was tense and mildly distended but not tender. Laboratory testing indicated an elevated white blood cell count (15.4 × 10^9^ cells/L), elevated lactic acid (3.9 mmol/L), reduced pCO_2_ (31 mmHg), reduced pO_2_ (46 mmHg), reduced bicarbonate (16 mmol/L), and red blood cells in urinalysis (>100 RBC/HPF). Her prothrombin time (PT) (68 s), international normalized ratio (INR) (7.9), and the activated partial thromboplastin time (aPTT) (140 s) were elevated. The patient stated that she was not on anticoagulation, but admitted to chronically, as well as acutely, smoking SC which she referred to as “K2 spice”.

**Fig. 2:**
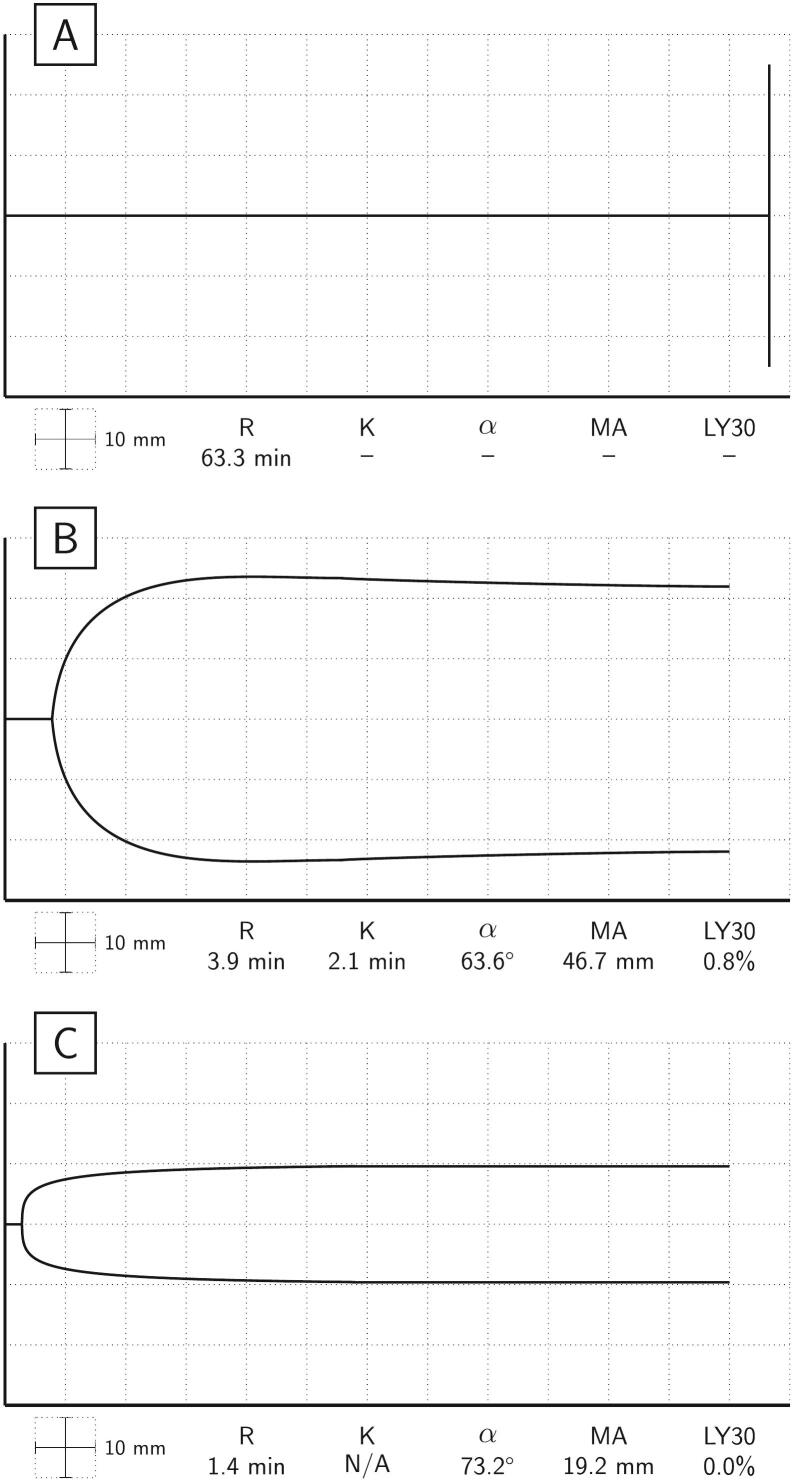
(A) Pre-resuscitation kaolin thromboelastography tracing is flatline. (B) Kaolin thromboelastography post operation reveals a much-improved tracing after administration of prothrombin complex concentrate, vitamin K, and fresh frozen plasma. (C) Thromboelastography functional fibrinogen post operation reveals a normal functional fibrinogen maximum amplitude.

SCAC due to contamination with brodifacoum was suspected since the INR was out of proportion to the trauma given her base deficit, lactic acid level, maintained mental status, and transient blood pressure response to resuscitation. Kaolin thromboelastography (TEG) taken on arrival to the emergency department demonstrated a flatline tracing consistent with excess anticoagulation (Fig. 2A). The patient was given multiple units of packed red blood cells (PRBCs), fresh frozen plasma (FFP), and platelets. After obtaining improved hemostasis with her blood pressure in the 110s mmHg systolic, her right hip was reduced. Due to the flatline TEG tracing, prolonged PT/INR and aPTT, and history of inhaling SC, it was decided to formally treat her for brodifacoum toxicity with 5000 units of PCC 70 units/kg and 10 mg of vitamin K intravenously. Additional units of PRBCs and FFP were also administered at this time. She was intubated and taken to the hybrid operating room for coiling of the hepatic vessels.

Immediately after embolization, a vertical midline abdominal incision released 3 L of free blood. The origin of bleeding was identified behind the liver, worse immediately left of the falciform ligament. During severe episodes of hypotension, the aorta was compressed manually. Considering the apparent retrohepatic injury, the falciform ligament was taken down. A complete vena cava avulsion between the liver and diaphragm was discovered. Given the patient's young age, alert mental status prior to intubation, lack of lactic acidosis, and relatively mild base deficit, the team opted for vena caval shunt insertion. The inferior vena cava was isolated and looped below the renal pedicle. Following mediastinotomy, a chest tube was placed connecting the right atrial appendage to the vena caval stump inferior to the vessel loops. This successfully allowed diversion of blood away from the area of transection.

The vena cava transection, including damage to the left hepatic vein at the vena caval junction, was repaired. Intraoperatively, the patient was given 40 units of FFP, 53 units of PRBCs, 10 units of single donor apheresis platelet concentrates, two 10-unit bags of cryoprecipitate, two 5000-unit doses of PCC (70 mg/kg), and two 1-mg doses of factor rVIIa (100 μg/kg). At the conclusion of the procedure, hypokinesis of the right ventricle and signs of diffuse bowel ischemia were noted. The mediastinum was closed, and the abdomen was left open with a vacuum-assisted closure.

Thirty minutes postoperatively, her coagulation screen showed a PT of 16 s, INR of 1.3, and aPTT of 20 s. Final thromboelastographic analysis normalized with a R time of 3.9 mins, K time of 2.1 mins, and α angle of 63.6°; however, it also revealed a low maximum amplitude of 46.7 mm and high lysis at 30 min of 0.8% (Fig. 2B). A TEG functional fibrinogen assay was within normal limits with a maximum amplitude of 19.2 mm (Fig. 2C). Despite improvement of the PT/INR, aPTT, and TEG, the patient remained hypotensive.

Postoperatively, her pupils were non-reactive, and she did not respond to painful stimuli or generate breaths over the set ventilator rate. Two and a half hours post operation, it was determined that the patient had sustained non-survivable injuries. She experienced brain death due to prolonged hypotension as a result of hemorrhagic shock where the bleeding had been exacerbated by brodifacoum.

## Discussion

The most significant aspect of our case is that this patient had a dramatically prolonged PT/INR and aPTT. It was clear that this was not solely due to trauma-induced coagulopathy for the patient was alert, had stable initial vital signs, and had relatively low base deficit and lactic acid. Even with this severe of an injury, such a profound coagulopathy would not be expected. SCAC contributed to the uncontrolled extent of hemorrhage at all stages after her injury, and thus, to her demise.

**Fig. 3:**
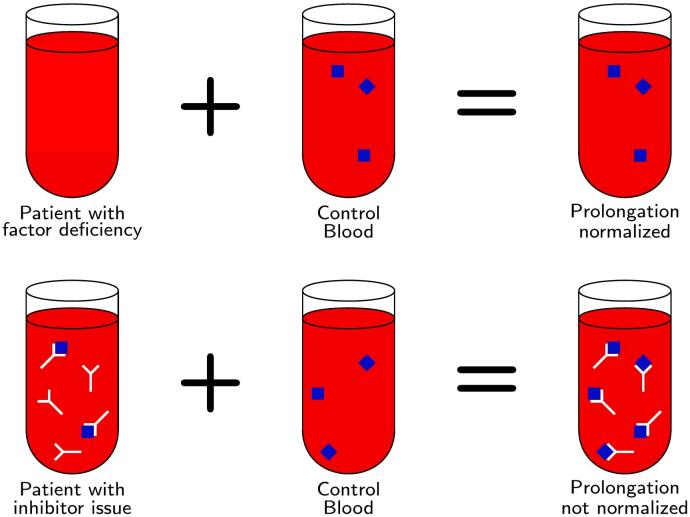
In a 1:1 mixing study, if the aPTT or PT prolongation corrects, the patient has a factor deficiency. If the prolongation does not correct, the patient has a factor inhibitor issue.

For any patient with unexplained prolongation of PT/INR and/or aPTT, it is important to perform classical 1:1 mixing studies. Correction of a 1:1 mixing study indicates that factor deficiency is the cause of the coagulation time prolongation rather than a specific factor inhibitor [5]. Brodifacoum antagonizes vitamin K epoxide reductase so that gamma carboxylation of factors II, VII, IX and X cannot take part in active coagulation, resulting in a prolonged PT/INR and/or aPTT [1,2]. The immediate 1:1 mixing study will reveal correction of the PT/INR and/or aPTT as the factors from the normal plasma will be used in forming a clot [5] (Fig. 3).

Given the recent increase in the use of SC, especially in the young patient population most likely to suffer severe traumatic injury [1,4,[6], [7], [8]], it would be prudent to assume that traumatologists will find more patients whose PT/INR and/or aPTT are out of proportion to the severity of the trauma. It will be important for traumatologists to determine if the patient has recent history of SC use and consult with hematologists to obtain mixing studies for these patients.

Our patient was able to confirm that she had a history of chronic and acute use of inhaled SC which no doubt contributed to her coagulopathy. Her PT/INR, aPTT, and TEG parameters corrected after the administration of PCC, vitamin K, and FFP. The patient's SCAC rendered her potentially survivable traumatic injuries to be lethal.

## CRediT authorship contribution statement

**Anthony V. Thomas:** Project administration, Visualization, Writing – original draft, Writing – review & editing. **Mackenzie L. Johnson:** Project administration, Writing – review & editing. **Anna M. Tincher:** Writing – review & editing. **Saniya Zackariya:** Writing – review & editing. **Hassaan Khan:** Writing – review & editing. **Uzma Rizvi:** Writing – review & editing. **Scott G. Thomas:** Investigation, Writing – review & editing. **Timothy W. Noveroske:** Investigation, Writing – review & editing. **Daniel H. Fulkerson:** Funding acquisition, Writing – review & editing. **Ernest E. Moore:** Supervision, Writing – review & editing. **Mark M. Walsh:** Conceptualization, Project administration, Supervision, Writing – original draft, Writing – review & editing.

## Declaration of competing interest

EEM has received research grants from Haemonetics Corp. Braintree, MA, outside the submitted work. The remaining authors declare that the research was conducted in the absence of any commercial or financial relationships that could be construed as a potential conflict of interest.

## References

[1] Kelkar A.H., Smith N.A., Martial A. (2018). An outbreak of synthetic cannabinoid-associated coagulopathy in Illinois. N. Engl. J. Med..

[2] Corke P.J. (1997). Superwarfarin (brodifacoum) poisoning. Anaesth. Intensive Care.

[3] Hui C.H., Lie A., Lam C.K. (1996). ‘Superwarfarin’ poisoning leading to prolonged coagulopathy. Forensic Sci. Int..

[4] Kircher M., Perez J. (2020). Brodifacoum poisoning linked to synthetic marijuana use in Wisconsin. WMJ.

[5] Kershaw G., Favaloro E.J., Lippi G. (2017). Hemostasis and Thrombosis: Methods and Protocols.

[6] Adams A.J., Banister S.D., Irizarry L. (2017). “zombie” outbreak caused by the synthetic cannabinoid AMB-FUBINACA in New York. N. Engl. J. Med..

[7] Feinstein D.L., Hafner J., van Breemen R. (2022). Inhaled synthetic cannabinoids laced with long-acting anticoagulant rodenticides: a clear and present worldwide danger. Toxicol Commun..

[8] Shymon S.J., Arthur D., Keeling P. (2020). Current illicit drug use profile of orthopaedic trauma patients and its effect on hospital length of stay. Injury.

